# Persistence Conditions of Institutional Entities: Investigating Copredication Through a Forced-Choice Experiment

**DOI:** 10.3389/fpsyg.2021.528862

**Published:** 2021-11-30

**Authors:** Elliot Murphy

**Affiliations:** ^1^Vivian L. Smith Department of Neurosurgery, McGovern Medical School, University of Texas Health Science Center, Houston, TX, United States; ^2^Texas Institute for Restorative Neurotechnologies, University of Texas Health Science Center, Houston, TX, United States; ^3^Department of Linguistics, University College London, London, United Kingdom

**Keywords:** copredication, polysemy, pragmatics, ship of theseus, persistence conditions, forced choice experiment

## Abstract

The conditions under which certain complex polysemous nominals can sustain coherent sense relations (informally, can “survive”) is investigated through a two-alternative forced choice experiment. Written scenarios were constructed which permitted copredication, through which multiple, semantically different sense types are associated with a single nominal. Participants were presented with two scenarios involving a polysemous nominal (e.g., *bank*, *city*) and had to select which scenario (and, hence, which combination of predicates) appeared to be the most prototypical, faithful realization of the nominal. In order to achieve this, an additional manipulation was added, such that the number of senses hosted by each forced choice was either equal (2 senses choice vs. 2 senses choice) or unequal (1 sense choice vs. 2/3 senses choice). In order to address certain concerns in the literature about prototypicality, a core question addressed was whether the institutional sense of the nominals strongly determined the option chosen by participants, or whether the number of senses more strongly predicted this. It was found that the best predictor of sense “survival” was not sense frequency, but rather sense complexity or approximation to the institutional sense.

## Introduction

In a recent discussion on the nature of consciousness, Chomsky (2018, p. 38) considers “one of the most ancient problems of philosophy: How can we cross the same river twice?” These are problems of “identity” and “individuation.” Relatedly, Collins (2017, p. 680) presents an example demonstrating how river can license copredication through it being a geographical feature, an abstract relation or a body of water:

“The Nile runs the length of Egypt and it serves as the most important trade route in the region as well as the source of irrigation for nigh-on all of Egypt’s crop production.”

This issue has been discussed mostly within the philosophical literature; witness Collins (2017, p. 686): “Thus, a group of people and a geographical area wildly dissociate in every conceivable sense save for them being referred to by *London*, say. We can kill the population of London, but not the area in south-east England. Equally, we can burn the city down while sparing the people, but rebuild the same city elsewhere, with a new population.”

It has been argued that these issues pertaining to the semantic diversity of apparently simple entities may relate to the theme of polysemy. Cognitive models of polysemy have suggested that vagueness, polysemy and homonymy represent “a cline of diminishing schematicity and increasing instances of salience” (Lewandowska-Tomaszczyk, 2007, p. 139), such that polysemous senses bear a lower degree of salience than homonymous meanings do, and vague words have an even lower degree of salience for a particular meaning. For instance, *student* is not polysemic with respect to the distinction between *young student* and *old student* (“I gave the book to a student but not to a student” cannot refer to an old and young student, respectively); it is vague and unmarked. In a similar way that one would typically say “Milton Keynes is close to London” and not “London is close to Milton Keynes,” due to proximation to a larger city being seen as a prototypical frame of geographical reference, it may be that there is an empirically detectable range of sense prototypicality in polysemy such that one sense may be seen as more essential to the polysemous nominal than others.

The main set of prototype effects are plotted in Table 1, loosely adapted from Lewandowska-Tomaszczyk (2007, p. 151) and modified to focus on the form of polysemy explored here. This shows how degrees of salience, clustering of senses, a lack of clear necessary and sufficient features, and variability in category membership are hallmarks of prototype effects. Table 1 plots effects relating to *extensions* and *intensions*, which are, respectively, entities referred to by the concepts and senses which the concepts are composed of. These effects can in turn be categorized based on whether they effect the salience of the sense (how clear and prominent it is in a given interpretation) or its discreteness (related to issues of demarcation).

**TABLE 1:** Main prototype effects and their mutual relationships.

	Extentionally	Intensionally
Salience	Differences of salience among senses	Clustering of senses into nominal representations
Discreteness	Senses can demarcate different entities	Absence of definitions in terms of necessary and sufficient features

As the framework depicted in the above table would predict, it has been found that people tend not to categorize objects using necessary and sufficient features, but rather do so by comparing their similarity to a prototype of the candidate category (Rosch, 1975, 1978). As such, when judging whether a cluster of senses in a given context constitutes a *school* or not, comprehenders will presumably compare this structure to their stored prototypical representation.

Yet, the main theoretical difficulty in discussing this topic surrounds the issue of what precisely constitutes the prototype. As Gries (2015, p. 473–474) notes, the prototypical sense of a word could be the most frequent, most salient, most concrete, or the earliest acquired sense, and it is furthermore likely that the criteria of prototypicality differs across nominal classes. As such, there seems little fundamental connection between prototype structure and models of polysemy; rather, the prototype is a representation likely independent of semantic or pragmatic processes (Murphy, 2016) and, the criteria for being a prototype likely differs across word classes and conceptual domains.

As Geeraerts (1989) also proposed, there are certain concepts (for example, INSTITUTION-related polysemous nominals) which may not be able to be defined through a set of necessary and sufficient features, and which exhibit a semantic structure which assumes the form of a set of clustered and overlapping interpretations. For example, “The school with large windows starts at 9 a.m. and has a strict headmaster and unruly students” contains a number of clustered senses being attributed to a single nominal. However, it may be the case that one of these senses is more salient and prototypical than the others, but intuition alone does not seem a powerful enough measure to expose this. As such, behavioral data is needed.

All of these effects are clearly present in a particular instantiation of polysemy known as *copredication*, whereby multiple, semantically different sense types are associated with a single nominal (“Lunch was delicious but was delayed”; “The newspaper that I held this morning has been sued”). Progressing on from recent research into the acceptability dynamics of copredication (Murphy, 2019, 2021a,b) and possible lower-level accounts (Murphy, 2018, 2020), I will investigate what I will term the *persistence conditions* of copredication, making close contact with the nature of prototypical copredications. The main research question addressed will be: What are the conditions under which the identity of a given entity can survive? This will act as a refined, controlled version of the classical Ship of Theseus paradox and the river paradox of Heraclitus. Crucially, the very notion of persistence conditions more readily lends itself to forms of polysemy involving copredication, since the notion of copredication is rooted in a sense of semantic conflict and incompatibility, rendering the construction of scenarios involving some aspect of competition between senses feasible.

In terms of focal predictions, the persistence conditions of these entities could be primarily determined by the number of senses being referred to in the discourse. This would suggest that sense number renders the ongoing representation of the entity salient, supporting its ultimate representational perseverance. Alternatively, one particular sense may more strongly predict how the object persists, such that, for example, the institutional sense of *school* determines its persistence, and not any other sense (e.g., a *school* might not be conceived as a building with an institution, but an institution with a building). One might relate these predictions to certain existing models of polysemy. For example, the Sense Enumeration Lexicon Hypothesis maintains that distinct senses of polysemous words like *school* are in fact represented as separate lexical entries, such that the persistence of any individual sense would be predicted not to be directly reliant upon any other sense, since these are lexically independent (supporting the sense number prediction) (Lehrer, 1990; Foraker and Murphy, 2012). On the other hand, the One Representation Hypothesis maintains, broadly speaking, that a word like *school* has multiple, underspecified representations connected to a single lexical entry, and would more directly be related to the sense type prediction (Frisson, 2009, 2015). More specifically, Löhr’s (2021) distinction between rich and thin semantic representations of polysemy (both of which are in accord with the One Representation Hypothesis) are relevant here. The thin view maintains that one specific sense forms the core meaning, around which other senses are clustered, while the rich view sees multiple senses forming distinct contributions to lexical meaning whilst still maintaining the existence of a single lexical entry. For more extensive discussion of polysemy storage and processing models (see Carston, 2016, 2019; Vicente, 2018; Ortega-Andrés and Vicente, 2019).

## Materials and Methods

### Materials

An online acceptability judgment experiment was carried out using Qualtrics^1^ and sourcing participants from Prolific Academic (prolific.ac). The purpose of the experiment was to present participants with scenarios involving a nominal that licenses copredication (e.g., *bank*, *city*) and ask them to make a forced choice to determine which outcome of the scenario (and, hence, which combination of predicates) appears to be the most prototypical, faithful realization of the nominal. In order to achieve this, an additional manipulation was added, such that the number of senses hosted by each choice was either equal (2 senses choice vs. 2 senses choice) or unequal (1 sense choice vs. 2/3 senses choice). All participants saw all trials across both types.

A core question of interest was whether the INSTITUTION sense strongly determined the option chosen by participants, or whether the number of senses predicted this. Addressing this question required balancing the number of scenarios in which the institution sense appeared either in isolation or with other senses. As such, in three scenarios the institution sense was isolated, in three scenarios it appeared with other senses while a different sense (e.g., PHYSICAL) was isolated, and the remaining six scenarios contained an equal number of senses.

The scenarios involved a two-alternative forced choice between qualified (licensed/acceptable) copredications of differing types, with participants being asked to choose *Which is the bank?* or *Which is the city?* 12 narratives were constructed, 6 of which presented two options exhibiting an equal number of senses, and the other 6 presented two options with an unequal number of senses. The narratives themselves exhibited all of the senses in the choices for participants. The central question which arises here is: What determines the more popular choice amongst participants? One possibility is *sense frequency*, i.e., participants will choose whichever option hosts the most frequent sense. Another possibility is *sense complexity*, with the most semantically complex sense (the one able to host the greatest number of senses and which is related to the greatest number of “core knowledge systems” or cognitive modules; Carey, 2009) determining true or prototypical objecthood for a given nominal.

Narratives of the following type were constructed (see Supplementary Appendix for full list). In the experiment, each narrative was followed by a choice: “Which is the X?”:

*Library*: A library catches on fire and is shut down. A new building across the street with self-service machines is built to help the public take out books. However, the employees of the original building protest and insist that the old library can simply be repaired, and refuse to hand over most of the books to the new building.

Choice: Old building—New building

Senses: Physical, People—Physical, Process

*Village*: The King of a medieval village becomes corrupt and so the folk stage a rebellion, burning down the whole place in the process. Taking the village’s original architects and main political leaders with him, the King relocates to a new site to accurately rebuild it. The village’s entire population, however, move to a different site and also bring with them one of the original architects who helps them rebuild.

Choice: King’s site—People’s site

Senses: Physical, Polity—Physical, Populace

Six fillers were also used to ensure participants were paying attention, with these narratives having clearer and more obvious answers. These were of the following kind:

*Sandwich*: John decides to make a sandwich. He slices it in half, begins to eat the first half, but then finds the bread very hard and difficult to swallow. He decides to take the second half and blend it into a smoothie.

Choice: First half—Second half

### Procedure

Scenarios were presented as single paragraph blocks, over a white background. Scenarios were presented in a random order. Below each paragraph, two options were presented corresponding to either choice, and participants were tasked with selecting their choice. After the presentation of the two-alternative forced choice, the screen was refreshed and participants were tasked with selecting one option from a 1–5 confidence metric, introduced by the question “How confident are you about your choice?” This allows differentiation between cases in which participants strongly believed in their choice, and cases when they were more ambivalent. Finally, to ensure that participants paid attention, they were forced to explain their reasons for selecting either option.

### Participants

79 native English speakers (average age: 36, range: 20–60, 60 female) took part in the experiment, sourced from Prolific Academic and having an approval rating on the site of at least 90%. Participants were paid £6 per hour, with the average finishing time being 10 min. This study was approved by the UCL Research Ethics Committee and participants consented immediately prior to the experimental procedure to their recording responses being used for academic research purposes.

## Results

### Confidence Scores

The average confidence score for the fillers was 4.2, and was lower for the experimental items (3.6). Confidence scores for the experimental items ranged from 3.01 (*city*) to 4.06 (*bank*), suggesting that the responses can be taken as an accurate and genuine reflection of the participant’s semantic intuitions. Confidence scores for experimental items can be found in Table 2.

**TABLE 2:** Average confidence scores across all nominals.

Nominal	Score (1–5)
Library	*3.75*
Village	*3.94*
Factory	*3.51*
City	*3.01*
University	*3.64*
Town	*3.71*
Church	*3.84*
Shop	*3.88*
Bank	*4.06*
Company	*3.29*
Province	*3.16*
School	*3.46*

### Sense Type Score

**FIGURE 1:**
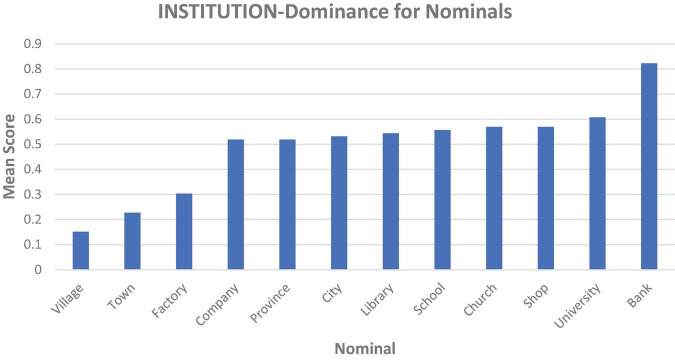
Average scores for all nominals, with the *Y*-axis representing percentage of Institutional sense selection (0.1 = 10%), where POLITY is subsumed as an institutional sense and narratives involving no explicit institutional sense were sorted such that the sense most approximate to INSTITUTION acted as an institutional proxy (e.g., POPULACE/EMPLOYEES is more semantically related to INSTITUTION than PHYSICAL).

Participants scored correctly on most fillers, and the minority of deviations (25/474 total responses) provided well-reasoned explanations. The results for the experimental narratives are plotted in Figure 1.

A multiple regression analysis was conducted using SPSS 24 with Sense Equality (Equal vs. Unequal) and Nominal (1–12 of the nominal list) as independent variables and Sense Type (Institution vs. Non-Institution) as the score. Sense Equality and Nominal significantly predicted Sense Type [*F*(2, 945) = 19.659, *p* < 0.001]. While Sense Equality added statistical significance to the prediction (*p* < 0.001), Nominal did not (*p* = 0.438). The average Sense Type score for the Unequal nominals was 0.59 (with 1 being 100% Institution sense chosen) and was 0.39 for the Equal nominals.

## Discussion

The results suggest that adding a greater degree of sense variability to each decision (e.g., Village #1 vs. Village #2) results in a more stable role for the institutional sense in determining objecthood. Participants were more willing to select a Non-Institution sense when the number of senses was matched across choices, but when the number of senses was unequal this seems to have exposed the possibility that the institutional sense was somehow more primary and essential, since when the number of senses is equal the (putative) primacy of the institutional sense would be harder for participants to detect, only becoming clear when they were forced to make a more stark choice between the institutional or non-institutional senses.

Comparing the present results to those from a previous frequency experiment (Murphy, 2019) allows us to determine whether the results were modulated by sense dominance. For *village* and *town*, PHYSICAL is the most frequent sense (based on results reported in Murphy, 2019, 2021a), which initially appears to predict the present results, yet participants made their decision based on the PEOPLE sense, not PHYSICAL, since both options hosted a PHYSICAL sense, and indeed both these decisions may have been influenced more by the POINT OF ORIGIN than by PEOPLE. Either way, it is difficult to determine whether frequency was the determining factor. PHYSICAL is also the dominant sense of *factory*, and two new locations were part of this narrative so POINT OF ORIGIN played no role, and both choices also hosted a PHYSICAL sense, but as with *village* and *town* the PEOPLE sense appears to have determined the outcome; that is, the sense semantically closest to INSTITUTION (and also the most abstract sense) determined the outcome. While the participant’s choices for *company*, *school* and *university* on average matched the dominant sense of these nominals (INSTITUTION), twice as many other nominals (*province, city, library, church, shop, bank*) deviated from this.

In conclusion, the best predictor of the results was not sense frequency, but rather sense complexity or approximation to INSTITUTION. It was reasonable to hypothesize that frequency would have had at least some influence, not least because the present experiment was explicitly oriented toward pitting two entities against each other, with prototypicality likely being one of the best guides for participants to make a judgment. Yet, even under these circumstances, sense frequency was not the determining factor.

It might also initially appear that persistence conditions could more easily be explored by giving participants stages of decomposition, such that parts of a city (its people, institutions, etc.) are removed one-by-one in a given scenario, with participants being asked “Is this a city?” every step of the way. However, since objects can exist with only a single polysemous sense persisting this proposal would likely result in all participants (rightfully) selecting “Yes” up to the point when the last sense remains. As such, not only would this design not speak to the question of *core, essential* senses of complex polysemous nominals, it would also likely result in all participants claiming that each nominal survived the full destructive process simply because such a process would necessarily be a “sense-by-sense” level of destruction, since this is the only level of granularity one can operate at (with the only other possibility being manipulating pragmatic factors such as the context that the object denoted by the nominal found itself in, in which case we are back to the original design of the present experiment).

One potential objection to the present study is that it did not control for pragmatic factors, such as the tendency for participants to “side” with the underdog against a putative antagonist, encouraging them to choose “People’s location” over “King’s location” for the *village* narrative. However, this seems unlikely since both *village* and *town* contain a clear “antagonist” (the corrupt King representing the INSTITUTION sense and the violent gangs representing the POPULACE sense) but this did not predict responses, since participants on average chose the (“good”) POPULACE-PHYSICAL senses for *village* but the (“bad”) POPULACE-PHYSICAL senses for *town*, even when the PHYSICAL sense of *town* was described in the narrative as being doomed for demolition. Likewise, the majority of participants opted for the “antagonist” PHYSICAL-INSTITUTION senses for *university* rather than the PHYSICAL-POPULACE senses. The notion of POINT OF ORIGIN therefore seems to play a role in individuating institutional entities like *town*, as it also does for *school*, *company* and *shop*—but not for *bank*, seemingly due to FUNCTION trumping POINT OF ORIGIN. For Pustejovsky (1995), these two notions of FUNCTION and POINT OF ORIGIN are, respectively, referred to as the TELIC and AGENTIVE Qualia roles. It appears that, much as how Pustejovsky originally claimed, different Qualia roles are foregrounded across different nominals based on context—although, going somewhat beyond this, the present experiment also suggests that the relations of these Qualia roles might exhibit a more robust, generalizable structure or hierarchy, such that the telicity of *bank* is foregrounded not simply due to context/narrative, but because of the internal structure of the senses which compose the (highly) polysemous nominal. This hypothesis ties in with Lang and Maienborn’s (2011, p. 719) interesting proposal that institutional nominals appear to have a common PURPOSE semantic feature (essentially Pustejovsky’s TELIC role), with the senses enveloping schools and banks and shops ultimately being centered on a core lexical meaning: “[A] legal entity that organizes purposeful events to be performed and/or received by authorized groups of persons in specific locations.” Nevertheless, it is remains possible that certain pragmatic factors were a confounding factor (e.g., narrative perspective; coherence relations), and future research should aim to more carefully examine this possibility.

## Conclusion

This experiment has shown that specific semantic and pragmatic factors enter into judgments about the persistence of polysemous entities in complex scenarios. These results provide support for a version of the One Representation Hypothesis of polysemous lexical representations, through which different senses of polysemous words can be navigated around a mereologically focal and essential representation. As such, Löhr’s (2021) thin view of polysemy representation appears to be supported, although the specific sense which forms the thin “core” seems to vary across nominals. Further research is required to more systematically relate Löhr’s (2021) model to psycholinguistic concerns. The present findings do not directly support Vicente’s (2017) claim that the INSTITUTION sense is necessary for the persistence of these nominal types, nor do they support (Arapini’s, 2013, 2015) belief that the multiple senses of institutional entities are “clustered in a symmetric structure” (2013, p. 35), since there is variability in spite of the very strong trend in INSTITUTION-dominance. While there may in fact be no such thing as a core, essential sense for any of the nominals discussed (with each nominal being a cluster of senses with pragmatic factors determining which one is brought to the fore), the results suggest that there is considerable variation in the level of INSTITUTION-dominance the sense-cluster of each nominal exhibits.

Future experiments involving a larger range of scenarios could introduce additional factors to test, such as sentence type/syntax, or the presence of coherence relations between components of the scenario. Addressing the issue of pragmatics, narrative frames could also be kept consistent across nominals; statistical power could be boosted by increasing the number of nominals; and all nominals could be presented under both equal/non-equal sense number conditions, to more directly test the extent to which variations across nominals impacts persistence.

## Data Availability Statement

All datasets generated for this study are included in the article/Supplementary Material.

## Ethics Statement

The study was approved by the Department of Linguistics Ethics Committee (LING-2013-3). Written informed consent to participate in the study was provided by all subjects.

## Author Contributions

The author confirms being the sole contributor of this work and has approved it for publication.

## Conflict of Interest

The author declares that the research was conducted in the absence of any commercial or financial relationships that could be construed as a potential conflict of interest.

## Publisher’s Note

All claims expressed in this article are solely those of the authors and do not necessarily represent those of their affiliated organizations, or those of the publisher, the editors and the reviewers. Any product that may be evaluated in this article, or claim that may be made by its manufacturer, is not guaranteed or endorsed by the publisher.

## Footnotes

1
qualtrics.com/uk

## References

[B1] Arapini’s A. (2013). Referring to institutional entities: Semantic and ontological perspectives. *Appl. Ontol.* 8 31–57. 10.3233/AO-130122

[B2] Arapinis A. (2015). Whole-for-part metonymy, classification, and grounding. *Linguist. Philos.* 38, 1–29. 10.1007/s10988-014-9164-6

[B3] Carey S. (2009). *The Origin of Concepts.* Oxford: Oxford University Press. 10.1093/acprof:oso/9780195367638.001.0001

[B4] Carston R. (2016). The heterogeneity of procedural meaning. *Lingua* 175-176 154–166. 10.1016/j.lingua.2015.12.010

[B5] Carston R. (2019). “Ad hoc concepts, polysemy and the lexicon,” in *Relevance, Pragmatics and Interpretation*, eds Scott K. Clark B. Carston R. (Cambridge: Cambridge University Press), 150–162. 10.1017/9781108290593.014

[B6] Chomsky N. (2018). “Mentality beyond consciousness,” in *Ted Honderich on Consciousness, Determinism, and Humanity*, ed. Caruso G. (London: Palgrave Macmillan), 33–46. 10.1007/978-3-319-66754-6

[B7] Collins J. (2017). The copredication argument. *Inquiry* 60 675–702. 10.1080/0020174X.2017.1321500

[B8] Foraker S. Murphy G. L. (2012). Polysemy in sentence comprehension: effects of meaning dominance. *J. Memory Lang.* 67 407–425. 10.1016/j.jml.2012.07.010 23185103 PMC3505093

[B9] Frisson S. (2009). Semantic underspecification in language processing. *Lang. Linguist. Compass* 3 111–127. 10.1111/j.1749-818X.2008.00104.x

[B10] Frisson S. (2015). About bound and scary books: the processing of *book* polysemies. *Lingua* 157 17–35. 10.1016/j.lingua.2014.07.017

[B11] Geeraerts D. (1989). Prospects and problems of prototype theory. *Linguistics* 27 587–612. 10.1515/ling.1989.27.4.587

[B12] Gries S. (2015). “Polysemy,” in *Handbook of Cognitive Linguistics*, eds Da̧browska E. Divjak D. S. (Berlin: de Gruyter Mouton), 472–490. 10.1515/9783110292022-023

[B13] Lang E. Maienborn C. (2011). “Two-level semantics: semantic form and conceptual structure,” in *Semantics*, eds Maienborn C. von Heusinger K. Portner P. (Berlin: De Gruyter), 709–740. 10.1515/9783110226614.709

[B14] Lehrer A. (1990). Polysemy, conventionality, and the structure of the lexicon. *Cognit. Linguist.* 1 207–246. 10.1515/cogl.1990.1.2.207

[B15] Lewandowska-Tomaszczyk B. (2007). “Polysemy, prototypes, and radial categories,” in *The Oxford Handbook of Cognitive Linguistics*, eds Geeraerts D. Cuyckens H. (Oxford: Oxford University Press), 139–169.

[B16] Löhr G. (2021). Does polysemy support radical contextualism? On the relation between minimalism, contextualism and polysemy. *Inquiry* 2021:1868329. 10.1080/0020174X.2020.1868329

[B17] Murphy E. (2016). Phasal eliminativism, anti-lexicalism, and the status of the unarticulated. *Biolinguistics* 10 21–50.

[B18] Murphy E. (2018). “Interfaces (travelling oscillations) + recursion (delta-theta code) = language,” in *The Talking Species: Perspectives on the Evolutionary, Neuronal and Cultural Foundations of Language*, eds Luef E. Manuela M. (Graz: Unipress Graz Verlag), 251–269.

[B19] Murphy E. (2019). “Acceptability properties of abstract senses in copredication,” in *Perspectives on Abstract Concepts: From Cognitive Processing to Semantic Representation and Linguistic Expression*, eds Bolognesi M. Steen G. (Amsterdam: John Benjamins), 145–165. 10.1075/hcp.65.08mur

[B20] Murphy E. (2020). *The Oscillatory Nature of Language.* Cambridge: Cambridge University Press. 10.1017/9781108864466

[B21] Murphy E. (2021a). *Linguistic Representation and Processing of Copredication.* Ph. D. thesis. London: University College London. 10.31234/osf.io/yubkz

[B22] Murphy E. (2021b). Predicate order and coherence in copredication. *Inquiry* 2021:1958054. 10.1080/0020174X.2021.1958054

[B23] Ortega-Andrés M. Vicente A. (2019). Polysemy and co-predication. *Glossa J. General Linguist.* 4:564. 10.5334/gjgl.564

[B24] Pustejovsky J. (1995). *The Generative Lexicon.* Cambridge, MA: MIT Press.

[B25] Rosch E. (1975). Cognitive reference points. *Cognit. Psychol.* 7 532–547. 10.1016/0010-0285(75)90021-3

[B26] Rosch E. (1978). “Principles of categorization,” in *Cognition and Categorization*, eds Rosch E. Lloyd B. B. (Hillsdale: Lawrence Erlbaum), 27–48.

[B27] Vicente A. (2017). What words mean and express: semantics and pragmatics of kind terms and verbs. *J. Pragmat.* 117 231–244. 10.1016/j.pragma.2017.07.007

[B28] Vicente A. (2018). Polysemy and word meaning: an account of lexical meaning for different kinds of content words. *Philosop. Stud.* 175 947–968. 10.1007/s11098-017-0900-y

