# Supplementary material for: Persistence Conditions of Institutional Entities: Investigating Copredication Through a Forced-Choice Experiment
Source: Front Psychol. 2021 Nov 30;12:528862. doi: 10.3389/fpsyg.2021.528862 (PMC8669508; doi:10.3389/fpsyg.2021.528862)
Supplement: Supplementary file 1 [file Data_Sheet_1.docx]

**Appendix**

*Experimental Narratives*

*Factory*: A factory goes out of business and so all the employees move to a new location to try and set up business. However, their new building is empty and they may not be able to perform their job. Meanwhile, in a third location some machines are set up to robotically perform factory tasks, but they lack any materials or managers.

Choice: Employee site – Machine site

Senses: Physical, Populace – Physical, Process

*City*: A city is burned down in an accidental fire. Its political leaders and half the population move to a new site, constructing a new style of buildings. The other half of the population, frustrated at this new style, move to a different site with the city’s original architects and faithfully construct the original style.

Choice: New style – Original style

Senses: People, Polity – People, Physical

*University*: The chancellors and managers of a university (St John’s University) decide to merge with a neighbouring university (St Catherine’s University), becoming a single university (St John & Catherine University). However, the students of both universities and most of the teaching staff reject the merger, and decide to continue teaching and studying in the original buildings, refusing to cross the boundaries set by the original two universities.

Choice: St John’s University – St John & Catherine University

Senses: Physical, Populace – Physical, Institution

*Town*: An old town is facing being demolished by the government. The population is mostly composed of thieves and corrupt, violent gangs, and they are strident in staying where they are, whereas the town’s council and legal institutions decide to agree to the government’s decision to move the town to a new location, rebuilding it faithfully. The population stay in the old, soon-to-be-demolished location, while the new town is being built.

Choice: Old town – New town

Senses: Physical, Populace – Physical, Institution

*Church*: A church catches on fire and is largely destroyed, and all the staff and most of the church's possessions are moved across the road to an abandoned building, which they gradually transform into a church through reconstruction. They continue to work in the new building but will most likely repair the old building at some point, since the new building only has a small number of physical features which they believe a church should have.

Choice: Old building – New building

Senses: Physical – Physical, People, Process

*Shop*: A bike shop goes out of business because of careless management. Frustrated, its employees move across town and begin to sell bikes in a new location. The owners of the original bike shop are soon forced out of their location because they cannot pay the rent, and are forced to move their headquarters to a new, cheaper building. They also have no employees to sell their bikes but retain the legal ownership of the firm’s identity.

Choice: Employee shop – Owner shop

Senses: Institution – Process, Physical

*Bank*: The electricity grid powering a bank is shut down, and the bank’s owners, employees and machinery are moved to a new building. The mechanics say they are unsure if they will be able to fix the original building’s electricity problems, and the future of the company is uncertain.

Choice: Original location – New location

Senses: Physical – Institution, Process

*Company*: An insurance company has a large number of employees in its building. For tax reasons, the company bosses decide to move their headquarters to a different location. This new location serves as the base for the company bosses, while the employees remain in the old building and perform the work.

Choice: Employee building – Headquarters

Senses: Physical, People – Institution

*Province*: An earthquake totally destroys a province and all its infrastructure. Thankfully, the human casualties are minimal. However, while the government decides to build a new province upstream along the river and successfully rebuilds the original buildings, the vast majority of the population take this as a chance to start afresh, and decide to build a province downstream along the river, reconstructing some of the original buildings but mostly starting anew.

Choice: Government province – Population province

Senses: Physical, Polity – Populace

*School*: A large fire destroys a school at night. The headteachers and government managers claim that there is not enough money to form a new school at a different site, even though this could be done fairly quickly, and so they decide to gradually rebuild the original school. However, the parents and approximately half the teachers decide that this will take far too long, leaving the children out of education at a crucial time for the students. They manage to raise enough money to rebuild the school at a new site, becoming a newly-formed school. A few months later, the original building has been rebuilt, and the government is confronted with the difficult challenge of assigning funding, and new parents are confronted with the difficult decision of choosing between schools.

Choice: Government school, New school

Senses: Institution – People, Physical
